# The Psychology of the “Red Back”

**Published:** 1912-03

**Authors:** J. C. Warbrick

**Affiliations:** Chicago, Ill.


For Texas Medical Journal.

### The Psychology of the "Red Back."

Almost every medical journal that is published possesses an individuality of some kind of its own that at once distinguishes it from all others. This may be in its size, color, style, or in the arrangement of its matter. Some have, perhaps, a more marked individuality than others in one or more ways that is at once noticeable.

The color of the cover of one journal may be mostly white; another may have the cover yellow, or green, while others may be red, or a combination of blue and black, white and black, or white and gold.

Of all the medical journals published, there is one that is distinct in its appearance from all the others at a glance. This journal is the "Red Back" (TEXAS MEDICAL JOURNAL), of Austin, Texas, the *guiding star* of the medical profession and its interests in Texas and the Southwest.

Now, the "Red Back" is not *yellow,* because it does not print news that is sensational. It is not green, because it does not get *bilious* even when a good many are behind in their subscriptions, and the editor occasionally has to take a jaunt to the San Marcos river for recreation. It is not white, because it does not claim to sail in a cloudless sky. It is not *blue,* because it is farther from the "blues" than the sun is from the earth. No; it is *Red,* because it indicates a good healthy color, a full one hundred per cent by the hemoglobin scale, with a normal specific gravity,—what every physician needs for himself and for his patients. *Red* indicates the blazing torch that beckons its subscribers onward and upward with Daniel at the wheel, guiding and keeping the machinery going while he steers the "Red Back" to greater heights and fields of usefulness.

J. C. WARBRICK, M. D.

Chicago, Ill.

